# Infectious shock after liposuction

**DOI:** 10.1186/s12879-022-07574-1

**Published:** 2022-07-15

**Authors:** Jinqiang Lu, Xiao Jiang, Hongyin Huang, Lingzhi Tang, Xinhui Zou, Haoran Mao, Hongwei Liu

**Affiliations:** 1 Department of Plastic Surgery, The First Affiliated Hospital of Jinan University, Huangpu West Road 613, Guangdong Province 510630 Guangzhou, People’s Republic of China; 2 Innovative Technology Research Institute of Tissue Repair and Regeneration, Key Laboratory of Regenerative Medicine, Ministry of Education, Huangpu West Road 613, Guangdong Province 510630 Guangzhou, People’s Republic of China

**Keywords:** Liposuction, *Clostridium perfringens*, Gas gangrene, Complications

## Abstract

**Background:**

Liposuction has become one of the most popular cosmetic surgeries in China. However, few studies have discussed infectious shock caused by *C. perfringens* as one of the causes of death after liposuction.

**Case presentation:**

A 24-year-old woman was brought to the emergency department (ED) of Guangzhou Chinese Overseas Hospital for treatment. The patient had undergone liposuction in her bilateral lower limbs two days prior. At the ED, the patient was unconscious, and had bilateral equal-sized (diameter, 6 mm) round pupils, no light reflex, a blood pressure (BP) of 71/33 mmHg, a heart rate of 133 bpm, and an SpO2 of 70%. She had bilateral limb swelling, extensive ecchymoses in her lower abdomen and bilateral thighs, local crepitus, blisters, weak pulses on her femoral artery and dorsalis pedis, high skin tension, and hemoglobin of 32 g/L. The patient was diagnosed with *Clostridium perfringens* infection, and she underwent debridement surgery and supportive treatment. But the patient’s BP could not improve. At 8:28 pm on the day of admission, the patient was declared clinically dead after the electrocardiograph showed a horizontal line and spontaneous respiration ceased.

**Conclusions:**

Failure to meet surgical disinfection and environmental standards may be the cause of infection of *C. perfringens* through wounds. Therefore, it is necessary to strengthen the environmental disinfection of the operating room, and standardize the sterile conditions of the operation staff and patients before and during operation. Liposuction surgery necrotizing fasciitis is a rare but fatal complications, especially if diagnosis delay, therefore it is critical for early diagnosis and treatment of gas gangrene.

**Supplementary Information:**

The online version contains supplementary material available at 10.1186/s12879-022-07574-1.

## Background

Liposuction is defined as the removal of localized subcutaneous fat deposits by suction and curettage or blunt cannulation for the cosmetic correction of obesity or other esthetic contour defects [1]. In China, liposuction can be performed in a hospital, a surgeon’s office, or a private cosmetic clinic. It is one of the most popular cosmetic surgical operations currently known worldwide [2]. However, with the increasing prevalence of liposuction, the occurrence of its complication is also increasing, such as postoperative infection, thromboembolic disease, skin irregularity, fat embolism, pulmonary edema, lidocaine intoxication, and intraabdominal visceral lesion, among others [3]. Although liposuction is a low-risk office-based procedure, there is still a life-threatening risk if infected. Therefore, analyzing different cases of liposuction can provide plastic surgeons with more information and experience about rescue plans performed after certain complications, helping them in their clinical practice.

Infection after liposuction is rare at less than 1% [4]. Some severe infections after liposuction can manifest as toxic shock syndrome, necrotizing fasciitis, cellulitis, staphylococcal abscess, and rapidly growing atypical mycobacteria; gas gangrene is relatively rare [5].

Several studies have reported infection after liposuction in the past, but only a few of these cases involved gas gangrene or infectious shock as one of the causes of death. The incidence of postoperative infection after liposuction is relatively low following standard and clean procedures, but postoperative infection is usually caused by *Staphylococcus aureus*, Streptococcus group A, and *Streptococcus pyogenes*. However, here we discuss a mortality from postoperative gas gangrene caused by *C. perfringens* contracted after liposuction, outlining the details of the rescue plan performed.

## Case presentation

A 24-year-old immunocompetent woman was admitted to the emergency department (ED) in a state of coma. The patient had liposuction done in her bilateral lower limbs two days ago, but the specific procedure done remains unknown. After her operation, the patient went shopping and had some street food. The patient then went home with no complications.

In the evening, the patient had swelling and pain in her right lower limb. She was given analgesic treatment, but it did not have any significant symptomatic relief.

Twelve hours after her operation, the patient visited the local hospital and was given tramadol for analgesia. But she still had no symptomatic relief, reporting persistent bilateral swelling, pain, and a poor spirit.

Sixteen hours after her operation, the patient visited a plastic and reconstructive surgery clinic and was administered analgesic and cooling treatment. At that time, the patient’s body temperature was 39.0 ℃. The patient was found to have scattered ecchymosis on her bilateral lower limbs with blisters.

Twenty-two hours after her operation, the patient visited the ED of Shenzhen Hospital with a body temperature of 37.1 °C, bilateral limb swelling, extensive ecchymoses on her lower abdomen and bilateral thighs, local crepitus, blisters, weak pulses on her femoral artery and dorsalis pedis, and high skin tension. Then patient accepted emergency CT and was started on cefamandole + ornidazole (anti-infective), intramuscular tetanus anti-toxin, fluid replacement, and dopamine to raise her blood pressure.

Twenty-eight hours after her operation, the patient became unconscious with an SpO2 of 87%. She was given an endotracheal intubation with a ventilator to assist her respiration. The patient’s vital signs improved to an SpO2 of 100% and a heart rate (HR) of 139 bpm with regular blood pressure monitoring. For further diagnosis and treatment, the patient’s families contacted an ambulance and the patient was sent to the ED of Guangzhou Overseas Hospital.

Thirty-one hours after her operation, the patient arrived at the ED of Guangzhou Chinese Overseas Hospital. Physical examination revealed unconsciousness, bilateral round pupils of equal size with a diameter of 6 mm, no light reflex disappeared, 71/33 mmHg BP (with dopamine support), a HR of 133 bpm, and an SpO2 of 70% with endotracheal intubation to assist respiration. Doctors in the ER started her on dopamine to maintain blood pressure. She was then admitted to Guangzhou Chinese Overseas Hospital with the following diagnosis: “(1) Finding the cause of lower limb infection accompanied by soft tissue infection; (2) Infectious shock; (3) Postoperative status after bilateral lower limb liposuction.”

**Fig. 1:**
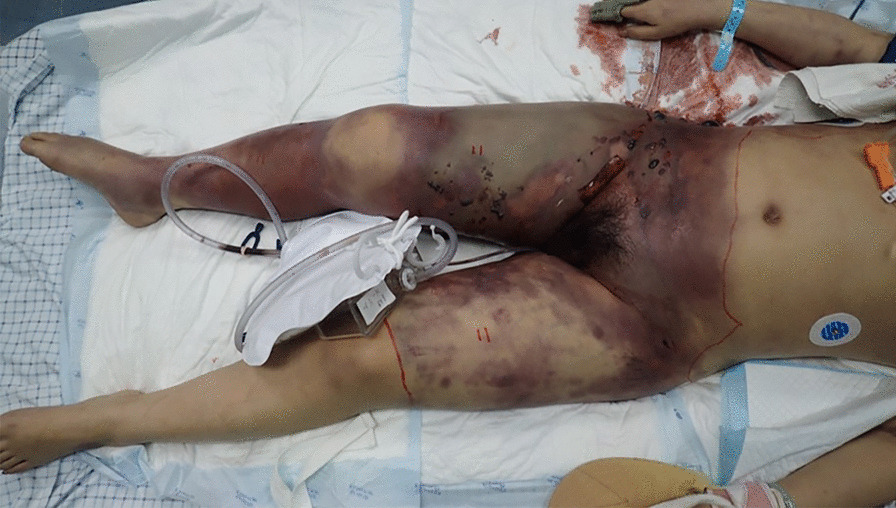
Extensive ecchymoses on abdomen, waist, butt, perineum, and bilateral lower limbs with blood blisters

Physical examination after admission revealed that she had extensive ecchymoses on her abdomen, waist, buttock, perineum, and bilateral lower limbs with blood blisters (Fig. 1). Bloody exudates were observed in areas with skin damage. Her bilateral lower limbs were highly swollen, especially in the right lower limb, in which the skin tension was high with local crepitus. A weak carotid artery pulse was palpable, but her peripheral artery pulses were unpalpable. Both her pupils were round and of equal size at a diameter of 5.0 mm, but she had no light reflex. She had an HR of 143 bpm with regular rhythm. Muscle tone in her bilateral upper limbs was at level 2, but no limb movements were observed in her bilateral lower limbs. No pathological reflexes were observed.

### Laboratory examination

Her blood panel showed High sensitivity C-reactive protein (HsCRP) at 197.23 mg/L, white blood cell (WBC) at 18.18 × 10^9^/L, monocyte percentage (MONO%) at 1.3%, eosinophil percentage (EOS%) at 0.1%, neutrophil (NEU#) at 9.82 × 10^9^/L, lymphocyte (LYM#) at 8.05 × 10^9^/L, red blood cell (RBC) at 2.13 × 10^12^/L, hemoglobin (HGB) at 37 g/L, hematocrit (HCT) at 11.3%, mean corpuscular volume (MCV) at 53.2 fL, mean corpuscular hemoglobin (MCH) at 17.6 pg, and red blood cell distribution width (RDW-CV) at 33.2%. Her coagulation functions showed an activated partial thromboplastin times (APTTs) of 110.1 s, a prothrombin time (PT) of 45.8 s, an international normalized ratio (INR) of 4.84, a fibrinogen degradation product (FDP) of 43.72 μg/mL, and a d-dimer (DD) of 8600 ng/mL. Finally, her myocardial infarction examination showed a creatine kinase-MB (CK-MB) of 118.0 ng/mL, a high sensitivity troponin-1 (HSTNI) of 0.669 ng/mL, and a brain natriuretic peptide (BNP) of 35,000 pg/mL (Additional file 1).

### Rescue process

The patient was unstable, had multiple organ dysfunction (MODS), was in a coma, and had weak spontaneous respiration. Her left pupil diameter was 6.0 mm while the right pupil diameter was 5.0 mm. The patient was intubated and was persistently hooked to a ventilator. She patient had circulatory failure and was therefore given high-dose noradrenaline (54 mg), high doses of intravenous blood products (RBC 11U, plasma 3000 mL, albumin 200 mL, and fibrinogen 2 g), volume expanders, and fluid replacement to rescue her symptoms. However, her BP still fluctuated greatly with the patient reaching critical condition.

**Fig. 2:**
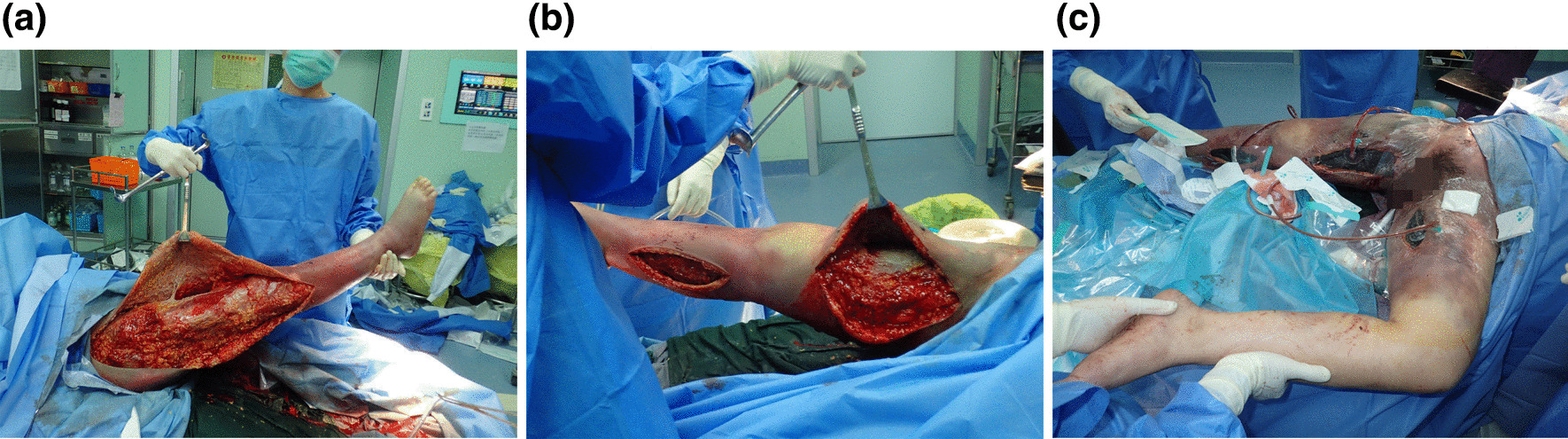
**a** Necrotic and devitalized fat tissue. **b** Subcutaneous, red, and foul-smelling effusion. **c** Vacuum drainage treatment

At 2:00 pm on the day of admission, the patient underwent debridement surgery and vacuum drainage in the Orthopedics Department. During the operation, doctors found a large amount of subcutaneous, red, and foul-smelling effusion, which was sent for bacterial culture. Necrotic and devitalized fat tissues were removed. The infection was mostly located outside the muscle (Fig. 2). However, the patient’s BP still could not be lifted even after giving her multiple vasopressors in large doses.

At 6:40 pm on the day of admission, her BP dropped to 58/20 mmHg, HR dropped to 86 bpm, her left pupil diameter remained at 6.0 mm, and her right pupil diameter increased to 5.5 mm. She still had no light reflex, and there were large amounts of exudates in her wounds.

The patient was given a 500 mL dextran injection to expand her volume, adrenaline to increase BP, and intravenous RBC and plasma. At 7:00 pm, the patient’s BP rose to 72/23 mmHg with an HR of 100 bpm. Succinyl gelatin injection, dextran, and albumin were given to her for volume expansion. Despite these efforts, her BP continued to decrease. Bacterial examination revealed positive results for *C. perfringens*. According to the bacterial examination, the antibiotic regimen was adjusted to given imipenem and cilastatin sodium 1 g Q12h, penicillin 800 × 10^5^iu Q8h and linezolid 600 mg Q12h.

At 8:28 pm,she still had no light reflex. What’s worse ,She no longer had palpable pulses, and her electrocardiograph monitoring showed a horizontal line. The patient had absence of spontaneous respiration and was declared to have clinical death.

The patient’s family agreed to have the patient undergo a postmortem examination. The recorded cause of death were as follows: (1) Extensive soft tissue infection (*C. perfringens* infection) and (2) Multiple organ dysfunction syndrome (DIC, kidney, liver, circulation, respiration, central nervous system). The recorded diagnosis of death were as follows: (1) Gas gangrene (*C. perfringens* infection), (2) Infectious shock, (3) Multiple organ dysfunction syndrome (DIC, kidney, liver, circulation, respiration, central nervous system), and (4) Postoperative liposuction in the bilateral lower limbs.

## Discussion and conclusion

In this case, the patient’s death was caused by gas gangrene. Two diagnoses should be differentiated from gas gangrene. The first is cellulitis, which can also be caused by *C. perfringens*. However, it typically does not involve muscle pain [6]. The second condition is necrotizing fasciitis. However, this is rarely caused by *C. perfringens* [7].

Gas gangrene is commonly caused by *C. perfringens* [8], which is a gram-positive, anaerobic pathogen commonly found in the gastrointestinal tracts of humans and animals. It produces extracellular enzymes and toxins (α and θ toxins) that act synergistically [9]. α toxins (phospholipase C) form pores on the cell membrane, leading to tissue and cell necrosis, exudate formation, and malignant edema [10]. In an in vitro experiment, it was shown that α toxins can inhibit the function of cardiac cells [11]. On the other hand, θ toxins break endothelial cells and cause hypoxia in the regional tissue. Other toxins have multiple enzyme activities that can further breakdown and liquify tissue, further spreading and exacerbating the infection.

*C. perfringens* multiply in different tissues and decompose carbohydrates and proteins, producing a large amount of gas that leads to tissue expansion [9]. X-rays and other imaging typically illustrate this subcutaneous gas build-up. Protein decomposition produces foul-smelling hydrogen sulfide, and the breakdown of vascular endothelial cells leads to regional edema. The combination of regional ischemia and other exotoxins further worsens tissue deterioration and necrosis. Macrophages and antibodies cannot reach necrotic tissue, leading to extensive tissue infection and exotoxin absorption, ultimately causing infectious shock. Some exotoxins directly invade the heart, liver, and kidneys, leading to locoregional necrosis or even multiple organ dysfunction. Under specific conditions, infections can spread to the whole body within one day, leading to shock or even death.

*C. perfringens* infection often occurs in patients with food poisoning, open injuries, and foreign bodies in the body. They may also occur after unsterile procedures, or in immunocompromised patients [13]. In this case, we speculate that the gateway of entry for *C. perfringens* in this patient was wound infection. The *C. perfringens* infection in this case may be attributed to a few reasons.

First, this operation was performed in a private cosmetic clinic. In recent years, the market for cosmetic surgery in China has been booming without strong regulation [13]. The hygiene problem of private cosmetic clinics in China has aroused great concern. In 2019, 86% of medical aesthetics entities in China were illegal, and 72% of medical aesthetics practitioners were non-licensed [14]. Therefore, the incidence of infection is high in these private cosmetic clinics. Some of these facilities may not conform to surgical standards for sterility, and C. perfringens may be carried and left in the operating room environment. In addition, preoperative patients may not have enough disinfection of the surgical site, allowing opportunistic pathogens-C. perfringens to enter the body through wound infection.

To decrease the risk of unsterile operations, all surgical procedures should follow standard surgical guidelines [15]. For example, the patient should be placed in a private ward, and an ultraviolet radiator and air sterilizer should be used to further sterilize the room after terminal disinfection.

Additionally, the patient went out for shopping after her operation and ate street snacks in the afternoon. After surgery, decreased immunity leads to an increased risk of infection. In addition, patients go shopping in a complex environment after surgery, which also increases the risk of infection. Although the skin incision of liposuction is small, the range of subcutaneous damage is large. Once infection occurs, it will provide a favorable environment for *C. perfringens* growth and reproduction. Therefore, after liposuction, the wound should be wrapped aseptically .The patient should be hospitalized for observation to avoid exposing the wound to unclean environment, preventing the infection of *C. perfringens* through the wound.

Although gas gangrene is a high-mortality disease, it can still be rescued given enough time [16]. To increase the possibility of survival, doctors should start treatment early, which includes active resuscitation, early application of antibiotics, and hyperbaric oxygen therapy [17]. Radical debridement, unobstructed drainage, and rinsing with a large amount of normal saline can effectively reduce the survival space of C. *perfringens* thereby reducing toxin absorption into the blood. The first-line antibiotic for gas gangrene is penicillin as high doses of penicillin and tetracycline can inhibit pyogenic infections. Meanwhile, hyperbaric oxygen therapy can inhibit the growth and proliferation of anaerobic bacteria, and to a large extent, narrow the area of necrotic tissue. Supportive therapy should also be considered throughout the treatment process, which includes giving a minimal number of blood transfusions, fluid replacements, vitamins, and high-protein and high-calorie diets. Analgesic, sedative, and antipyretic treatments should also be administered if necessary.

In addition to these treatment options, a vaccine for *C. perfringens* has been explored in recent years. However, most of the work on these vaccines and the toxins of *C. perfringens* have only involved animals [18]. We hope that we can also benefit from the work on animal *C. perfringens* vaccine and accelerate the development of *C. perfringens* vaccine for humans.

Liposuction is becoming increasingly popular in China. However, most medical aesthetics entities and practitioners in China are illegal or unlicensed, which is very risky because their operating rooms and procedures are unlikely to meet surgical standards, creating conditions for bacterial infection through the wound. This might be responsible for the *C. perfringens* infection-related death in this case. Gas gangrene is not a common cause of postoperative liposuction infection under standard surgical procedures, and treatment with gas gangrene should be initiated early with supportive treatment. In addition, education related to care after liposuction is critical. Patients should be told to rest in bed .Doctors and other practitioners should monitor the patient’s postoperative status and provide appropriate advice when needed.

## Supplementary Information

**Additional file 1: Table S1: **Laboratory examination which is abnormal:the redArabic numerals indicate the higherresults;the blue Arabic numerals indicate the lowerresults.

## Data Availability

All data generated or analysed during this study are included in the published article.

## Abbreviations

ED: Emergency Department
BP: Blood pressure
SpO2: Blood oxygen saturation
CT: Computer tomography
HR: heart rate
ER: Emergency room
HsCRP: High sensitivity C-reactive protein
WBC: White blood cell
MONO%: Monocyte percentage
EOS%: Eosinophil percentage
NEU#: Neutrophil
LYM#: Lymphocyte
RBC: Red blood cell
HGB: Hemoglobin
HCT: Hematocrit
MCV: Mean corpuscular volume
MCH: Mean corpuscular hemoglobin
RDW-CV: Red blood cell distribution width
APTTs: Activated partial thromboplastin times
PT: Prothrombin time
INR: International normalized ratio
