# Supplementary material for: Infectious shock after liposuction
Source: BMC Infect Dis. 2022 Jul 15;22:617. doi: 10.1186/s12879-022-07574-1 (PMC9287851; doi:10.1186/s12879-022-07574-1)
Supplement: Supplementary file 1 — Additional file 1: Table S1: Laboratory examination which is abnormal:the redArabic numerals indicate the higherresults;the blue Arabic numerals indicate the lowerresults. [file 12879_2022_7574_MOESM1_ESM.docx]

| Laboratory examination | result | Reference Values |
| --- | --- | --- |
| High sensitivity C-reactive protein (HsCRP) | 197.23 mg/L | 0-8mg/L |
| white blood cell (WBC) | 18.18*10^9^/L | 3.5*10^9^-9.5*10^9^/L |
| neutrophil (NEU#) | 9.82*10^9^/L | 1.8*10^9^-6.3*10^9^/L |
| monocyte percentage (MONO%) | 1.3% | 3%-10% |
| eosinophil percentage (EOS%) | 0.1% | 0.4%-8% |
| hemoglobin (HGB) | 37 g/L | 115-150g/L |
| activated partial thromboplastin times (APTTs) | 110.1 s | 28-44s |
| a prothrombin time (PT) | 45.8 s | 11-16s |
| fibrinogen degradation product (FDP) | 43.72 ug/mL | 0-5ug/mL |
| prothrombin time international normalized ratio (PTINR) | 4.84 | 0.81-1.36 |
| d-dimer (DD) | 8600 ng/mL | 0-500ng/mL |
| creatine kinase-MB (CK-MB) | 118.0 ng/mL | 0-3.4 |
| high sensitivity troponin-1 (HSTNI) | 0.669 ng/mL | 0-0.016ng/mL |
| brain natriuretic peptide (BNP) | 35000 pg/mL | 0-125pg/mL |

*Table1.*Laboratory examination which is abnormal:the red Arabic numerals indicate the higher results;the blue Arabic numerals indicate the lower reslults.
